# SLC39A7, regulated by miR-139-5p, induces cell proliferation, migration and inhibits apoptosis in gastric cancer via Akt/mTOR signaling pathway

**DOI:** 10.1042/BSR20200041

**Published:** 2020-02-28

**Authors:** Yanting Zhang, Jie Bai, Wangli Si, Shanshan Yuan, Yijun Li, Xiaolu Chen

**Affiliations:** 1 Department of Gastroenterology, Xi’an Central Hospital, Xi’an, Shaanxi 710003, China; 2 Department of Respiratory and Critical Medicine, Xi’an Central Hospital, Xi’an, Shaanxi 710003, China

**Keywords:** gastric cancer, miR-139-5p, proliferation, SLC39A7

## Abstract

As a zinc transporter, SLC39A7 (zip7) is vital in intestinal epithelial self-renewal, and recent studies suggested that SLC39A7 was related to cancer progression. Whereas, little is known about the role of SLC39A7 in gastric cancer (GC). In the present study, qRT-PCR analysis demonstrated that SLC39A7 mRNA level was increased in both GC tissues and cell lines. Overexpressing SLC39A7 boosted cell proliferation and migration, while inhibited apoptosis in GC. It was also found that si-SLC39A7 suppressed Akt/mTOR pathway and activation of Akt/mTOR pathway reversed the effects of si-SLC39A7 on GC development. Through prediction website, we found that SLC39A7 was directly regulated by miR-139-5p. miR-139-5p mimic had adverse effects on SLC39A7 expression and influence in the GC cell proliferation, migration and apoptosis by Akt/mTOR signaling pathway, while miR-139-5p inhibitor showed opposite effects. To conclude, our studies showed that SLC39A7 was negatively regulated by miR-139-5p. Besides, SLC39A7 positively regulated GC development through Akt/mTOR signaling pathway. These results indicate that SLC39A7 may be a candidate target gene for GC treatment.

## Introduction

Gastric cancer (GC) is one of the most common malignancies occurring in the digestive tract. Each year, more than 700,000 people die of GC worldwide and there are nearly 1 million new cases of GC. Furthermore, the relative 5-year survival rate for GC is less than 30% in most countries. Although surgery is a potential treatment for GC, unfortunately, only a few patients with GC have access to surgical treatment due to the high proportion of advanced tumors at the onset. Besides, the prognosis of patients with GC is still poor [1]. In this case, the discovery of the molecular mechanism influencing the progression of GC is urgently needed to provide new methods and means for the treatment of GC.

Zinc plays an important role in multiple biological processes, including DNA synthesis, mitosis, as well as differentiation [2]. Accumulating studies demonstrated that zinc was related to cell proliferation, invasion and apoptosis in various human cancers [3]. SLC39A7 (ZIP7) belongs to LIV-1 subfamily of zinc transporters which ensure the homeostatic maintenance of zinc via influencing the storage and redistribution of intracellular zinc [4]. SLC39A7 could be promoted by phosphorylation via protein kinase casein CK2 which is related to cell proliferation, migration, apoptosis and mitosis [5,6]. It has been proved that the phosphorylation of SLC39A7 conserves residues could lead to AKT phosphorylation, tyrosine kinases activation and ERK1/2 signaling pathway [6]. The knockdown of SLC39A7 has an adverse effect on cell proliferation, migration and invasion in cervical cancer [7]. In gastric tumor model, it was reported that SLC39A7 expression was remarkably up-regulated [8,9], while the function and mechanism of SLC39A7 in GC is still not clear.

MicroRNAs (miRNAs), endogenous noncoding regulatory RNAs with 17–25 nucleotides, participate in post-transcriptional gene regulation. They could cause multiple mRNA degradation or translational repression by targeting the complementary sequences in 3′-untranslated regions (3′-UTRs) of mRNAs. Accumulating researches reported that the expression of miRNAs was different between cancerous tissues and normal counterparts, like GC [10–13]. Furthermore, miRNA target genes to have effects on cancer cells development [14,15], which makes them tumor suppressors or oncogenes during cancer occurence and development [16]. miR-139-5p was down-regulated in human cancers, including GC cells [17], parathyroid carcinoma [18], endometrial serous adenocarcinoma [19], hepatocellular carcinoma [20] and breast cancer [21], in which miR-139-5p exhibited anti-oncogenic and anti-metastatic effects [22–26]. In GC, miR-139/Jun functions as a negative feedback loop for GC development which made miR-139 a feasible therapeutic target for GC treatment [27].

In this research, it was found that SLC39A7 expression was remarkedly promoted in GC tissues and cell lines. Besides, the present study firstly demonstrated that SLC39A7 was repressed by miR-139-5p and affected proliferation, migration and apoptosis of GC cells by Akt/mTOR pathway and this discovery may provide GC treatment with novel theoretical foundation.

## Materials and methods

### Patient tissues and cell transfection

Tissue samples and adjacent non-tumor tissues were gathered from 36 preoperative GC patients from 2010 to 2015 at Xi’an Central Hospital. Before surgery, none of the GC patients had radiotherapy or chemotherapy. And informed consent was gained from each patient and healthy control. All the tissue samples were identified by clinical pathologist. The research was performed in accordance with the World Medical Association Declaration of Helsinki. Informed consents were gained from each patient and healthy control.

Human GC cell lines HGC-27, SGC-7901, MKN-28 and MGC-803 (ATCC, Manassas, VA) were cultured in RPMI-1640 medium with 10% fetal bovine serum, 100 U/ml penicillin and 100 mg/l streptomycin. When the confluence grew to 80–90%, the cells were digested with trypsin and then transported to a 60-mm cell culture dish. The transfection of pcDNA3.1, pcDNA3.1-SLC39A7, si-RNA, si-SLC39A7, mimic NC, miR-139-5p (miR-139-5p mimic), inhibitor NC and miR-139-5p inhibitor were carried out through Lipofectamine 3000 (Invitrogen).

### Cell proliferation assays

Cells (5000/well) were maintained in 96-well plates for 24 h, 20 μl MTT solution (5 mg/ml) was added to each well and cultured for another 4 h. Next, culture was terminated and the medium was carefully removed. A total of 150 μl DMSO was added to each hole, and the absorbance was detected at the wavelength of 570 nm after oscillating on the shaking table at low speed for 10 min.

### Wound-healing assay

Cells (8 × 10^4^/well) were plated in 24-well plates and the cell monolayer was scraped using a sterile pipette tip. Then, cells were washed with PBS three-times and cultured in medium without serum for 1 h until cell recovery. The healing at 0 and 24 h was recorded. And the cell migration distance was assessed by Image-Pro Plus Analysis software (Media Cybernetics, Inc., Rockville, MD, U.S.A.).

### Cell apoptosis analysis

Cells were collected and 500 μl binding buffer was used to resuspend these cells. Cell suspension was incubated with 5 μl Annexin V-FITC in dark. Then, 50 μg/ml PI was added and flow cytometry (FACScan®; BD Biosciences) was used to analyze the apoptosis of cells.

### Dual luciferase reporter assay

The prediction website Starbase (http://starbase.sysu.edu.cn/index.php) was recruited to predict the putative miRNA which targeted SLC39A7. And dual luciferase reporter assay was applied to confirm the prediction result. First, the wild-type or mutant 3′-UTR of SLC39A7 was amplified and cloned into the vector psiCHECK-2 to construct luciferase reporter plasmids (WT SLC39A7 or MUT SLC39A7, respectively). Then, 293 T cells (1 × 10^4^/well) were co-transfected with WT SLC39A7 or MUT SLC39A7 and miR-139-5p or miR-NC via Lipofectamine 2000 for 48 h. And the Dual-Luciferase Reporter Assay kit (Promega Corp., Madison, WI, U.S.A.) was employed to detect the luciferase activity.

### Quantitative real-time PCR

Cells were harvested, the total RNA was isolated from tissues and cells using TRIzol reagent (Invitrogen, Carlsbad, CA, U.S.A.). Then, RNA was reversely transcribed into cDNA by the cDNA synthesis kit (ABI, U.S.A.) and quantitative real-time PCR was performed by a two-step quantitative RT-PCR experiment. GAPDH was recruited as the housekeeping reference. The comparative *C*_t_ method was used to assess the data. The primers used in this assay were: SLC39A7: 5′-CTGGAGCGGTGAGAATGAGAGG-3′ and 5′- ACTGGTGGGAGAAAGGAAACTGG-3′; GAPDH: 5′-GGACCTGACCTGCCGTCTAG-3′ and 5′-GTAGCCCAGGATGCCCTTGA-3′.

### Western blotting

Proteins were extracted with radio immunoprecipitation assay (RIPA) buffer (Cell Signaling Technology, U.S.A.). Fifty micrograms of protein extractions were separated using SDS/PAGE electrophoresis and then transferred to polyvinylidene fluoride (PVDF) membranes (Millipore, U.S.A.). The membrane was blocked with 5% skim milk powder for 2 h. Then the corresponding primary antibody was added and maintained overnight at 4°C. The membrane was washed with 1× TBST for three-times and incubated with the horseradish peroxidase–conjugated secondary antibody for 1 h. At last, ImageJ software was used for gray-scale analysis of the bands and relative quantitative analysis of protein expression levels.

### Statistical analysis

GraphPad Prism5 software was used to process and plot the result data, and SPSS 18.0 software was used to conduct paired *t* test or one-way ANOVA analysis for the difference comparison. All data were presented as the mean + SD. *P*<0.05 was considered statistically significant.

## Results

### SLC39A7 expression was higher in GC tissues and cell lines

**Figure 1:**
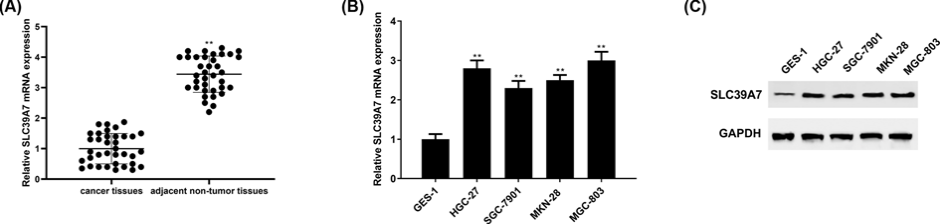
SLC39A7 is highly expressed in GC (**A**) qRT-PCR was used to detect SLC39A7 mRNA expression in GC tissues. (**B**) SLC39A7 mRNA and (**C**) protein level in GC cells were analyzed via qRT-PCR and Western blot. **P<0.01 *vs* normal tissues or GES-1 cells.

qRT-PCR was employed to analyze SLC39A7 mRNA expression in cancer tissues and matched adjacent non-tumor tissues from 36 GC patients as well as GC cell lines and normal cell lines. The results demonstrated that SLC39A7 was obviously higher expressed in tissues of GC patients than that of non-tumor tissues (Figure 1A). Besides, both SLC39A7 mRNA and protein expression level were up-regulated in GC cell lines (HGC-27, SGC-7901, MKN-28 and MGC-803) compared with human normal gastric mucosa cells line GES-1 (Figure 1B,C).

### SLC39A7 promoted cell proliferation and migration, and decreased apoptosis of MGC-803 and HGC-27

**Figure 2:**
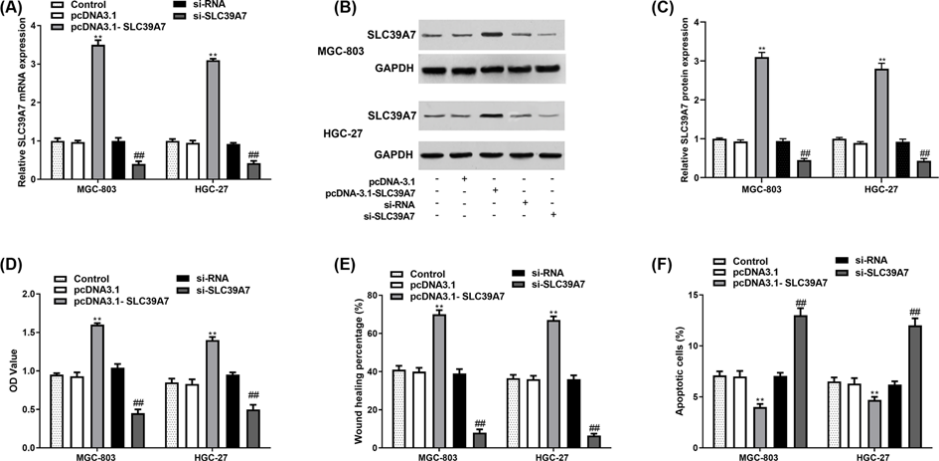
SLC39A7 promoted GC cell proliferation and migration while inhibiting cell apoptosis MGC-803 and HGC-27 cells were cultured and transfected with si-RNA, si-SLC39A7, pcDNA3.1 or pcDNA3.1-SCL39A7. (**A–C**) Transfection efficiency of pcDNA3.1-SLC39A7 and si-SLC39A7 were evaluated by qRT-PCR and Western blot. (**D,E**) Cell proliferation and migration were evaluated by MTT assay and wound-healing assay. (**F**) Cell apoptosis was evaluated by apoptosis analysis. **P<0.01 *vs* pcDNA3.1 and ^##^*P*<0.01 vs si-RNA.

To investigate the role of SLC39A7 in GC development, SLC39A7 was overexpressed by pcDNA3.1-SLC39A7 and down-regulated by si-SLC39A7, respectively (Figure 2A–C). MTT assay and wound-healing assay results demonstrated that compared with the control group, increased expression of SLC39A7 remarkedly promoted cell proliferation and migration of MGC-803 and HGC-27 cells, and si-SLC39A7 suppressed cell proliferation (Figure 2D) and migration (Figure 2E). While the cell apoptosis was inhibited by si-SLC39A7 and elevated by pcDNA3.1-SLC39A7 (Figure 2F).

### Akt/mTOR signaling pathway participated in the function of SLC39A7 on GC development

**Figure 3:**
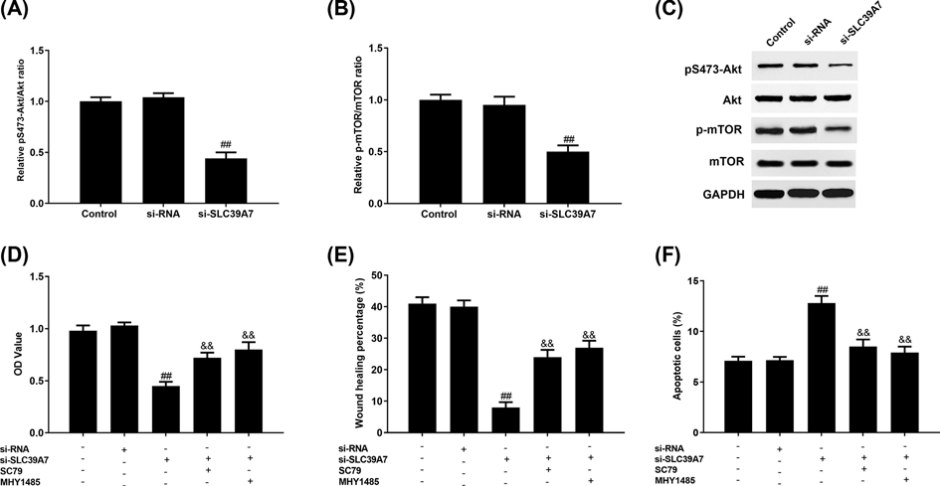
SLC39A7 affects GC cells proliferation, migration and apoptosis through Akt/mTOR pathway HGC-27 cells were transfected with si-RNA or si-SLC39A7 and treated with SC79 or MHY1485. pS473-Akt/Akt (**A,C**) and p-mTOR/mTOR (**B,C**) protein expression in HGC-27 cells was assessed by Western blot. (**D**) MTT assay, (**E**) wound-healing assay and (**F**) apoptosis assay were recruited to analyze the proliferation, migration and apoptosis of HGC-27 cells with si-SLC39A7 or si-RNA transfection and SC79 or MHY1485 treatment. ^##^*P*<0.01 vs si-RNA and ^&&^*P*<0.01 *vs* si-SLC39A7.

To gain a better understanding the regulatory mechanism of SLC39A7 in GC, we assessed whether SLC39A7 down-regulation influences the Akt/mTOR signaling pathway which is often aberrantly boosted in various human cancers and affects cell proliferation and apoptosis. Western blot results demonstrated that pS473-Akt (Figure 3A,C) and p-mTOR (Figure 3B,C) protein expression levels were significantly reduced in HGC-27 with si-SLC39A7 transfection. Futhermore, we found that both Akt activator, SC79 and mTOR activator, MHY1475 promoted cell proliferation (Figure 3D) and migration (Figure 3E) while inhibiting cell apoptosis (Figure 3F) compared with the si-SLC39A7 group without SC79 treatment.

### miR-139-5p directly targets SLC39A7 and inhibits its expression in MGC-803 and HGC-27

**Figure 4:**
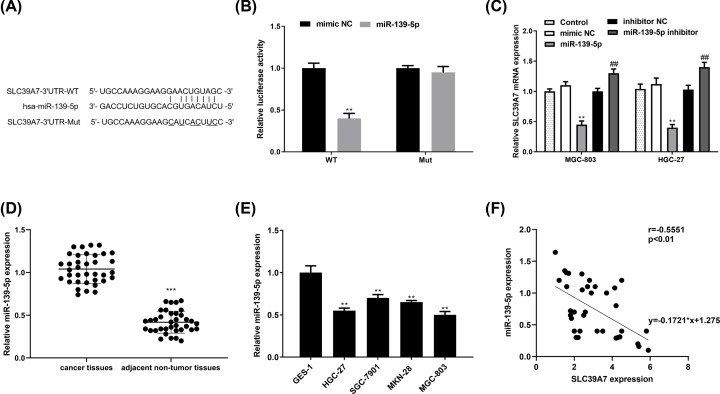
miR-139-5p targets SLC39A7 directly (**A**) The putative binding sequence of miR-139-5p in wild-type and mutant SLC39A7-3′UTR. (**B**) The relative luciferase activity with wild-type or mutant SLC39A7-3′UTR in HGC-27 cells transfected with the miR-139-5p or miR-NC were analyzed. (**C**) qRT-PCR was applied to assess SLC39A7 mRNA expression in miR-139-5p or miR-139-5p inhibitor transfected group and respective NC group. (**D,E**) mRNA levels of miR-139-5p in gastric tissues and cell lines were detected via qRT-PCR. (**F**) Spearman’s correlation analysis was recruited to explore the correlation between miR-139-5p and SLC39A7 mRNA level. **P<0.01 vs miR-139-5p and ^##^*P*<0.01 vs miR-139-5p inhibitor.

It was reported that 50–60% of all human genes were regulated by miRNAs [28] which are vital in various biological processes, including cell proliferation, migration and invasion [29]. Therefore, to gain a better understanding of SLC39A7 mechanism, Starbase (http://starbase.sysu.edu.cn/index.php) was recruited to explore whether there was an miRNA which could affect SLC39A7 expression. After selection and looking up related references, miR-139-5p was picked up for further research (Figure 4A). And luciferase report assay result showed that high miR-139-5p expression evidently inhibited the luciferase activity of pGL3- SLC39A7 3′-UTR WT but not the Mut (Figure 4B). To further confirm this relationship, we transfected miR-139-5p or miR-139-5p inhibitor and their controls into HGC-27 cells. It turned out that miR-139-5p mimic suppressed SLC39A7 mRNA expression while miR-139-5p inhibitor promoted SLC39A7 mRNA expression (Figure 4C). The qRT-PCR results demonstrated that miR-139-5p mRNA levels were significantly lower in gastric tissues and cell lines than the normal group (Figure 4D,E). Furthermore, Spearman’s correlation analysis indicated that miR-139-5p and SLC39A7 expression levels in OS tissues were correlated inversely (Figure 4F).

### MiR-139-5p inhibited Akt/mTOR pathway by targeting SLC39A7 in HGC-27 cell

**Figure 5:**
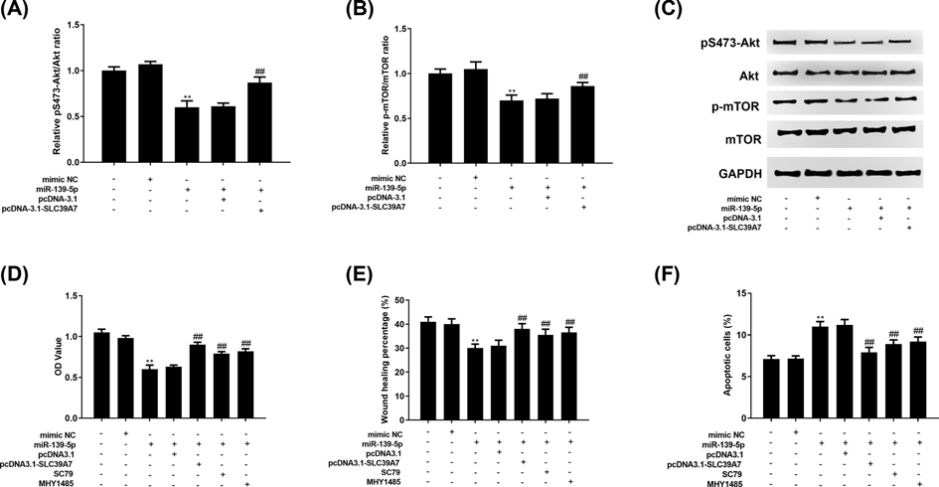
SLC39A7 mediated-Akt/mTOR pathway is involved in the miR-139-5p regulated cell proliferation, migration and apoptosis of GC HGC-27 cells were co-transfected with mimic inhibitor or miR-139-5p mimic and pcDNA3.1 or pcDNA3.1-SLC39A7 with SC79 or MHY1485 treatment. (**A–C**) The protein expression of pS473-Akt and p-mTOR was evaluated by Western blot. The cell proliferation (**D**), migration (**E**) and apoptosis (**F**) were analyzed via MTT assay, wound-healing assay and apoptosis assay. **P<0.01 vs mimic NC, ^##^*P*<0.01 vs miR-139-5p or miR-139-5p + pcDNA-3.1.

We next assessed the potential mechanism of miR-139-5p regulated GC proliferation, migration and apoptosis. The results demonstrated that both pS473-Akt and p-mTOR protein expression were decreased by miR-139-5p and increased by co-transfection of miR-139-5p and si-SLC39A7 (Figure 5A–C). Then, MTT, wound-healing and apoptosis assay results demonstrated that miR-139-5p curbed HGC-27 cell proliferation (Figure 5D) and migration (Figure 5E) while pcDNA3.1-SLC39A7 co-transfection and SC79 and MHY1485 treatment would reverse this tendency. The results of cell apoptosis (Figure 5F) were opposite.

## Discussion

SLC39A7 is essential for the vigorous proliferation of transit-amplifying cells and sustaining intestinal stem cells stemness [30]. Overexpressed SLC39A7 is beneficial for the growth and invasion of tamoxifen-resistant MCF-7 cells [31]. Similarly, SLC37A7 knockdown evidently curbs the proliferation and invasion of colorectal cancer cells [32]. Here we found that SLC39A7 expression was significantly higher in tissues and cells of GC than that of the normal group. It was reported that SLC39A7/ZIP7 was beneficial for intestinal epithelial self-renewal through resolving ER stress [30]. Furthermore, knockdown of SLC39A7 expression could result in the decrease in cell growth and the increase in cell apoptosis in colorectal cancer [32]. In accordance, we verified that in GC, SLC39A7 had the ability of inducing cell proliferation and migration while inhibiting cell apoptosis.

It was reported that the persistent activation of JAK/STAT3 [33], PI3K/AKT [34], MEK/ERK [35], Wnt [36] signaling in most cancer lesions leads to numerous genetic and epigenetic alterations. As a zinc transporter, SLC39A7 plays an important role in activating tyrosine kinase, leading to aggressive and invasive phenotypes of cancer cells [31,37]. Besides, SLC39A7 is demonstrated to regulate zinc-mediated tyrosine kinase signaling, which might make it to be positively associated with tumor progression [37]. It was reported that the silencing of SLC39A7 suppressed the zinc-induced activation of EGFR, IGF-1R, as well as Src [31], which transmit signals to PI3K/AKT/mTOR signaling pathways. PI3K/Akt/mTOR pathway aberrant is common in gastric carcinoma which regulates tumor occurence and progression, such as those in proliferation and in apoptosis. AKT has effects on cancer cell survival by influencing Bcl-2, p53, NF-κB and PTEN [38,39]. Furthermore, the dysregulation of PTEN/PI3K/AKT signaling interaction with the Wingless-INT pathway causes epithelial–mesenchymal transition, leading to cancer stem cell-phenotype and poor prognosis [40]. Akt/ERK-p53 signaling pathways kinase could activate the human telomerase which is a decisive factor contributing to the tumorigenesis [41–43]. While numerous mechanisms of tumor inhibition are still unknown, there are various small molecule inhibitors which target PI3K/Akt/mTOR pathway have been explored in clinical trials of gastric carcinoma [44]. Our present study demonstrated that PI3K/Akt/mTOR pathway was one of downstream targets of SLC39A7 and could be activated by SLC39A7 [45].

miR-139-5p was viewed as a tumor suppressor in hepatocellular carcinoma first [46]. Following researches demonstrated that suppressing miR-139-5p expression was beneficial for cell proliferation, migration and invasion, and had adverse effects on cell apoptosis in many types of cancers [46–51]. It was reported that miR-139-5p made colorectal cancer cells sensitive to 5-FU by suppressing NOTCH-1 expression [52]. Suppressed miR-139-5p could curb the proliferation, migration and invasion of non-small-cell lung cancer cells [53]. While up-regulated miR-139-5p suppressed aerobic glycolysis, as well as proliferation, migration and invasion of hepatocellular carcinoma cells [54]. In accordance with former research [17], we found that miR-139-5p was down-regulated in GC tissues and cell. And miR-139-5p inhibited SLC39A7 expression to affect GC cell proliferation, migration and apoptosis. Besides, miR-139-5p was reported to suppress the tyrosine phosphorylation of IRS1, phosphorylation of p85 subunit of PI3K, as well as serine phosphorylation of Akt [55]. In agreement, it was found that through curbing SLC39A7 expression, miR-139-5p inhibited Akt phosphorylation while Akt and mTOR activator, cooperate positively with miR-139-5p inhibitor. Thus, it was speculated that miR-139-5p may affect GC cell proliferation, migration and apoptosis through SLC39A7/Akt/mTOR axis.

In conclusion, the present study offered a novel insight into SLC39A7 function and mechanism in GC. We first demonstrated that SLC39A7, controlled by miR-139-5p though Akt/mTOR pathway, contributed to GC cell proliferation, migration and inhibited cell apoptosis. These explored effects and mechanism of SLC39A7 may be beneficial for paving theoretical basis for GC treatment.

## Abbreviations

Akt: protein kinase B
CK2: casein kinase 2
EGFR: epithelial growth factor receptor
ER: endoplasmic reticulum
GAPDH: glyceraldehyde-3-phosphate dehydrogenase
GC: gastric cancer
IGF-1R: the IGF-1 receptor
IRS1: insulin receptor substrate 1
miRNA: microRNA
mTOR: mammalian target of rapamycin
NC: Negative control
PI: propidium lodide
PI3K: Phosphatidylinositol-3-kinase
qRT-PCR: quantitative reverse transcription-polymerase chain reaction
5-FU: 5-fluorouracil
3′-UTR: 3′-untranslated region
